# Health-related quality of life and social constraints among Chinese breast cancer patients: a cross-sectional study

**DOI:** 10.1186/s12955-021-01871-0

**Published:** 2021-10-12

**Authors:** Chunying Cui, Lie Wang, Xiaoxi Wang

**Affiliations:** 1 Department of Social Medicine, School of Public Health, China Medical University, No. 77 Puhe Road, Shenyang North New Area, Shenyang, 110122 Liaoning People’s Republic of China; 2 Medical Basic Experimental Teaching Center, China Medical University, No. 77 Puhe Road, Shenyang North New Area, Shenyang, 110122 Liaoning People’s Republic of China

**Keywords:** Health-related quality of life, Social constraints, Social support, Social stigma, Chinese, Breast cancer

## Abstract

**Background:**

Most research studying social constraints has been performed among Caucasian or Asian American breast cancer (BC) patients, but few studies have evaluated social constraint levels and explored the effect of social constraints on the integrative health-related quality of life (HRQOL) of Chinese BC patients. Therefore, our study aimed to examine the association of social constraints with HRQOL among Chinese women with BC.

**Methods:**

This was a cross-sectional study of 136 Chinese women diagnosed with BC in Liaoning Province, China, from December 2020 to May 2021. Questionnaire information contained HRQOL, social constraints, social support, social stigma, and sociodemographic and clinical characteristics. Multiple linear regression was used to analyse the association of psychological factors with HRQOL.

**Results:**

The mean score of FACT-B was 96.05 (SD = 18.70). After controlling for potential confounders, social constraints (*Beta* =  − 0.301, *P* < 0.001) and social stigma (*Beta* =  − 0.241, *P* = 0.001) were negatively associated with HRQOL and social support (*Beta* = 0.330, *P* < 0.001) was positively associated with HRQOL, which explained 44.3% of the variance in HRQOL.

**Conclusions:**

The findings of the current study suggest that Chinese BC patients’ HRQOL needs to be enhanced after treatment. Social constraints have a strong association with HRQOL. Intervention strategies focusing on less personal disclosure should be considered to avoid social constraints and improve HRQOL among Chinese patients with BC.

## Introduction

Breast cancer (BC) is the most frequently diagnosed cancer and the leading cause of cancer death in the vast majority of countries. According to the most recent GLOBOCAN publication, the five most frequent cancers in Chinese women are breast, lung, colorectum, thyroid and stomach [1]. It was estimated in 2020 that the age-standardized (world) incidence of BC was 39.10/100,000 in China [2]. Furthermore, there are approximately 416,371 new BC cases and 117,174 deaths per year among Chinese women [2]. Due to early screening, early diagnosis and better treatment, patients with BC have a long survival time after diagnosis. Studies have reported that breast-conserving surgery and adjuvant radiotherapy in early BC patients result in 5-year overall survival rates of > 80% in China [3, 4]. As BC patients live longer, concerns about quality of life have increased. Hence, it is urgent and significant to evaluate and improve the health-related quality of life (HRQOL) of BC patients after treatment.

At present, BC treatment involves state-of-art biomedical therapy but can fail to address HRQOL issues, particularly emotional well-being and social/family well-being after diagnosis and treatment. Doege et al. conducted a study to compare disease-free BC patients’ HRQOL to that of non-cancer female controls and found that long-term BC survivors suffer significantly lower social, emotional, functional, physical well-being, fatigue, insomnia, and dyspnoea than female population controls [5]. However, a study conducted by Bower et al. indicated that many BC patients have relatively healthier lifestyles and found a new meaning to their lives in the long term [6]. In addition, long-term survivors reported similar or improved HRQOL levels based on exercise interventions compared to controls without BC [7]. Some sociodemographic and clinical characteristics among BC patients affect their HRQOL, including age [8], socioeconomic status (SES) [9], cancer stage [10] and treatment methods [11]. Moreover, an increasing number of investigators have focused on studying sociopsychological factors related to HRQOL among BC patients [5–8, 12, 13].

Most evidence has shown that low social support and high social stigma are importantly and significantly associated with impairments in HRQOL among BC patients [9, 13–15]. Social support refers to a combination of perceiving assistance available from family, friends and other significant relationships in times of need [16]. Based on the theory of the “stress buffering hypothesis” [17], social support can enhance individuals’ well-being and health by acting as a “direct agent”. In addition, it can act as an “antecedent factor” to increase individuals’ positive coping styles and psychological empowerment, which thus has a positive effect on mental health and HRQOL. For example, Strayhorn SM thought that positive social support has a positive association with HRQOL in BC patients [15]. In other words, social support is a positive psychological resource to evaluate BC patients’ HRQOL. Stigma is defined as feelings of isolation, degradation, criticism and rejection during personal experience and a social process that affects psychological, physical and social adjustment [18]. Indeed, previous studies have found that people with stigma have worse health outcomes than their non-stigmatized counterparts [19, 20]. A growing number of studies have examined stigma associated with cancer as cancer is perceived more negatively in society than some other diseases [21]. Cancer treatment leaves psychological and physical scars, such as stigma, surgical scars and hair loss [22]. BC patients with perceived stigma may avoid social relationships, which may result in an interpersonal stressor [23]. More importantly, perceived stressors may influence HRQOL among BC patients.

There is some evidence that social constraints have a strong association with decreased HRQOL and increased emotional distress among women with BC [9, 24, 25]. Social constraints are defined as the objective existence and subjective perceptions of social conditions, which hinder individuals from disclosing or modifying the ways of their disclosure of illness-related feelings or concerns [26]. Based on social cognitive processing theory [26, 27], individuals’ cognitive assimilation or accommodation of cancer experience can be facilitated by emotional disclosure (talking with empathic and supportive others, providing reappraisal, validation and meaning-making); cognitive processing thereby contributes to improving mental adjustment to cancer within a receptive social environment. When cancer patients experience unsatisfying interpersonal factors (others’ withdrawal, criticism and denial), an opposing effect occurs. Additionally, social constraints negatively affect healthy behaviours, including self-examination among BC patients [27], adherence to self-care activities among diabetes patients [28], and exercise [29]. However, most research studying social constraints has been performed among Caucasian or Asian American BC cancer patients, and few studies have evaluated social constraint levels and explored the effect of social constraints on integrative HRQOL among Chinese BC patients living in mainland China. There is only a study conducted by Yeung et al. [30] to explore the adverse effect of social constraints on functional well-being among BC patients living in Shandong Province, China. Owing to cultural differences, Chinese emotional disclosure is difficult to practice. Namely, Chinese people are more likely to value emotional suppression to avoid disturbing the interpersonal harmony that Chinese society emphasizes, whereas people from Western cultures tend to express their emotions due to the independent culture [31, 32]. Therefore, our study aimed to examine the association of social constraints with HRQOL among Chinese women with BC.

## Methods

### Participants

The present study included 136 Chinese women diagnosed with BC in Liaoning Province, China, from December 2020 to May 2021. Their average age was 48.05 (SD = 10.24). Among the recruited participants, 77.2% lived in cities, and 66.2% had an educational background of senior middle school or above. The majority of participants (85.3%) were married/cohabiting, and more than 50% of participants had a family per capita monthly income above 3000 (CNY). Of them, 23.5% had a time since diagnosis above two years. All patients had initial stage I (36%) or stage II (64%); about 50% of patients subsequently developed distant metastases after stopping treatment. Details of sociodemographic and clinical information are presented in Table 1.

**Table 1:** Sociodemographic and clinical information among Chinese BC patients

Variables	N	Percent (%)
Age	136	48.05 (10.24) (30–78)*
*Residence*		
Rural area	31	22.8
City	105	77.2
*Education background*		
Junior middle and below	46	33.8
Senior middle school	63	46.3
College and above	27	19.9
*Marriage status*		
Single/divorced/widowed/separated	20	14.7
Married/cohabited	116	85.3
*Employment*		
Yes	60	44.1
No	37	27.2
Retirement	39	28.7
*Family per capita monthly income (CNY)*		
Less than 3000	58	42.6
3000–5000	43	31.6
More than 5000	35	25.7
*Children*		
No	10	7.4
Yes	126	92.6
*Religious faith*		
No	126	92.6
Yes	10	7.4
*Period*		
Normal	36	26.5
Disorder	16	11.8
Amenorrhea	84	61.8
*Smoking*		
No	129	94.9
Yes	7	5.1
*Drinking*		
No	106	77.9
Yes	6	4.4
Quitting	24	17.6
*Time since diagnosis*		
Half year or below	47	34.6
Half to 2 years	57	41.9
More than 2 years	32	23.5
*Distant metastasis*^a^		
No	65	47.8
Yes	71	52.2
*Cancer stage*^b^		
I	49	36.0
II	87	64.0

^a^Subsequent development of distant metastases

^b^Disease stage at diagnosis

^*^Mean (SD) (range)

### Procedures

This study had a cross-sectional design. All participants were recruited at Shengjing Hospital of China Medical University. The inclusion criteria for the present study were as follows: (1) participants were diagnosed with BC; (2) they received treatments, such as breast-conserving surgery and/or adjuvant radiotherapy; and (3) they could communicate fluently in Chinese and voluntarily participate in the survey. The exclusion criteria were as follows: (1) other severe diseases (other cancers) and (2) a history of psychiatric or cognitive disorders. In total, 212 patients met our inclusion criteria, and 150 participants provided written informed consent to participate in our study. After obtaining informed consent for the present study, investigators distributed the questionnaires to the participants. In addition, medical information was collected from their clinical records. Finally, 136 valid questionnaires were recovered after excluding 14 questionnaires with poorly answered responses. The effective response rate of the questionnaires was 90.7%.

### Measures

#### Health-related quality of life (HRQOL)

The Chinese simplified version of the Functional Assessment of Cancer Therapy-Breast (FACT-B) Version 4.0 was used to assess BC patients’ HRQOL [33]. The scale contains 36 items to measure BC patients’ physical (PWB, seven items), emotional (EWB, six items), social/family (SFWB, seven items), functional well-being (FWB, seven items), and concerns about BC (BCS, nine items) [33, 34]. Each item was scored on a five-point scale from 0 (not at all) to 4 (extremely). According to a previous study [14, 35], the FACT-B total score = PWB + SWB + EWB + FWB + BCS. A higher score reflects better HRQOL. In our study, the aim was to examine the effect of social factors (social constraints, social support and social stigma) on the integrative health-related quality of life (HRQOL) among Chinese BC patients. Therefore, we mainly discuss the associations of social factors with HRQOL based on the total score of FACT-B.

#### Social constraints

The current study used the 15-item Social Constraints Scale (SCS-15) to test BC patients’ social constraints in the past month [36]. You and Lu translated the SCS-15 from English to Chinese [24], and the scale has been confirmed to have good reliability and validity among Chinese American BC patients [9, 25]. The scale aims to measure the frequency with which individuals diagnosed with cancer feel socially constrained when discussing their cancer-related thoughts from their spouse (family or friends for patients without a spouse). Two items (tell you not to worry so much about your health; tell you to try not to think about the cancer) were deleted due to low item-total correlations (*r* = 0.188–0.235) compared to other items (*r* = 0.474–0.757), which is consistent with a previous study [37]. Therefore, there are 13 items in the present study, and each item is rated on a four-point scale from 1 (never) to 4 (often). A higher score indicates a higher frequency of experiencing social constraints.

#### Social support

The Chinese version of the Multidimensional Scale of Perceived Social Support (MSPSS) was used to measure the level of social support in BC patients [38] and has been widely used in other diseases [39]. The scale comprises 12 items, and the rating was made on a seven-point scale from 1 (very strongly disagree) to 7 (very strongly agree), with a higher score indicating sufficient support from family, friends or significant others.

#### Social stigma

The Chinese version of the Social Impact Scale (SIS) [40] was used to evaluate the level of stigmatization among patients with BC and has been confirmed to have good reliability in Chinese populations [39]. The scale contains 24 items and adopts a four-point scale from 1 (very strongly disagree) to 4 (very strongly agree), with a higher score indicating severe social stigma.

#### Sociodemographic and clinical characteristic

Sociodemographic characteristics were included in a set of self-administered questionnaires. In addition, medical information was collected from their clinical records. Participants’ sociodemographic and clinical information was collected, including age, residence, educational background, marital status, employment, income, children, period, smoking, drinking, religious faith, time since diagnosis, cancer stage (disease stage at diagnosis) and distant metastasis (subsequent development of distant metastases).

### Statistical analysis

First, Pearson correlation analysis was conducted to analyse the correlation between HRQOL and the continuous variables. T-tests and ANOVA were performed to examine the difference between HRQOL and the classification variables. Next, multivariable linear regression analysis was conducted to identify significant factors of HRQOL among the relational factors. In linear regression, HRQOL was regarded as a dependent variable, and social constraints, social support and social stigma were regarded as independent variables. In Model 1, social constraints were entered. Then, social support and stigma were added to Models 2 and 3, respectively. Finally, an adjusted Model 4 was performed to control for potential confounders according to univariate analysis (*P* < 0.25). A *P value* less than 0.05 was considered statistically significant (two-tailed) in the linear regression model.

SPSS for Windows (22.0) was used to conduct all the statistical analyses in the present study.

## Results

### Reliability of assessment scales

All scales presented good internal consistency in the present study: Cronbach’s alpha coefficient = 0.890 for FACT-B, 0.909 for SCS-15, 0.964 for MSPSS and 0.955 for SIS.

### Level of continuous variables

The average age of the participants was 48.05 (SD = 10.24) and ranged from 30 to 78. The total FACT-B scores ranged from 46 to 133, with a mean score of 96.05 (SD = 18.70). The mean scores of social constraints, social support and social stigma were 23.06 (SD = 8.38), 61.11 (SD = 15.49) and 47.99 (SD = 12.94), respectively (Table 2).

**Table 2:** Scores of continuous variables

Variables	Mean	SD
FACT-B total score	96.05	18.70
Social constraints	23.60	8.38
Social support	61.11	15.49
Social stigma	47.99	12.94

FACT-B total score = PWB + SWB + EWB + FWB + BCS

### Risk factors for HRQOL

Table 3 presents the relationships between HRQOL and each variable. BC patients’ HRQOL was possibly related to the following variables: patients who had children, religious faith, normal period, short time after diagnosis, and non-smoking status had better HRQOL (*P* < 0.25). Social constraints (*r* =  − 0.512, *P* < 0.01) and stigma (*r* =  − 0.434, *P* < 0.01) were negatively correlated with HRQOL; social support (*r* = 0.498, *P* < 0.01) was positively associated with HRQOL.

**Table 3:** Relationship among HRQOL and univariable

Variables	FACT-B total score	*P*
	Mean (SD)	
Age^c^	–	0.732
Social constraints^c^	–	< 0.01
Social support^c^	–	< 0.01
Social stigma^c^	–	< 0.01
*Residence*	–	0.774
Rural area	96.90 (21.11)	
City	95.80 (18.03)	
*Education background*		0.690
Junior middle and below	94.11 (19.52)	
Senior middle school	97.08 (17.70)	
College and above	96.96 (19.96)	
*Marriage status*		0.777
Single/divorced/widowed/separated	94.95 (22.22)	
Married/cohabited	96.24 (18.12)	
*Employment*		0.571
Yes	97.73 (17.78)	
No	93.59 (21.79)	
Retirement	95.79 (17.04)	
*Family per capita monthly income (CNY)*		0.483
Less than 3000	94.05 (19.87)	
3000–5000	98.60 (17.79)	
More than 5000	96.23 (17.88)	
*Children*		0.052
No	85.00 (20.67)	
Yes	96.93 (18.33)	
*Religious faith*		0.251
No	96.57 (18.64)	
Yes	89.50 (19.18)	
*Period*		0.232
Normal	100.47 (19.16)	
Disorder	96.38 (21.82)	
Amenorrhea	94.10 (17.76)	
*Smoking*		0.095
No	96.67 (18.73)	
Yes	84.57 (14.81)	
*Drinking*		0.279
No	94.85 (18.15)	
Yes	105.50 (18.53)	
Quitting	99.00 (20.84)	
*Time since diagnosis*		0.189
Half year or below	92.57 (19.30)	
Half to 2 years	99.26 (18.53)	
More than 2 years	95.44 (17.65)	
*Distant metastasis*^a^		0.430
No	94.72 (19.40)	
Yes	97.27 (18.08)	
*Cancer stage*^b^		0.973
I	95.98 (18.48)	
II	96.09 (18.92)	

^a^Subsequent development of distant metastases

^b^Disease stage at diagnosis

^c^Correlation analysis

FACT-B total score = PWB + SWB + EWB + FWB + BCS

Table 4 reports the results of the multivariable linear regression analysis. In Model 1, social constraints (*Beta* =  − 0.512, *P* < 0.001) were significantly and negatively associated with HRQOL and explained 25.7% of the variance in HRQOL. In Model 2, social support (*Beta* = 0.374, *P* < 0.001) was significantly and positively associated with HRQOL, while social constraints (*Beta* =  − 0.396, *P* < 0.001) were still negatively related to HRQOL. Additionally, the linear combination of variables in Model 2 explained 38.0% of the variance in HRQOL. In Model 3, social support (*Beta* = 0.375, *P* < 0.001) was found to positively impact HRQOL, but social stigma (*Beta* =  − 0.258, *P* = 0.001) was negatively related to HRQOL. Social constraints (*Beta* =  − 0.280, *P* < 0.001) were still significantly and negatively associated with HRQOL. The linear combination of variables in Model 3 explained 42.9% of the variance in HRQOL. Finally, confounders included in Model 4 were children, religious faith, period, smoking, and time since diagnosis. Social constraints (*Beta* =  − 0.301, *P* < 0.001), support (*Beta* = 0.330, *P* < 0.001), and stigma (*Beta* =  − 0.241, *P* < 0.001) were significantly associated with HRQOL, which explained 44.3% of the variance in HRQOL.

**Table 4:** Possible related factors of HRQOL

Variables	Model 1		Model 2		Model 3		Model 4	
	Beta	*P*	Beta	*P*	Beta	*P*	Beta	*P*
Social constraints	− 0.512	< 0.001	− 0.396	< 0.001	− 0.280	< 0.001	− 0.301	< 0.001
Social support			0.374	< 0.001	0.375	< 0.001	0.330	< 0.001
Social stigma					− 0.258	0.001	− 0.241	0.001
Children (no/yes)							0.120	0.083
Religious faith (no/yes)							− 0.119	0.071
Smoking (No/Yes)							− 0.016	0.819
Period^a^							− 0.082	0.229
Time^b^							0.092	0.167
Adjusted *R*^2^	0.257		0.380		0.429		0.443	

^a^period (normal, disorder, amenorrhea)

^b^Time (half year or below, half to 2 years, more than 2 years)

## Discussion

The mean FACT-B score was 96.05 (SD = 18.70) in Chinese women with BC. The level of overall HRQOL in the current study was lower than that in Americans in BC studies that adopted the same measurement tool [41–43]. In addition, Lu et al. found that Chinese BC patients reported lower HRQOL than their American counterparts [35]. Lu et al. suggested that Chinese BC patients undergoing more aggressive treatment or taking drugs may be likely to experience more adverse side effects [35]. Additionally, strategies of symptom control may be different. Despite the fact that there are affordable and advanced treatments to obtain better cancer care for Chinese BC patients, our results showed that patients with BC still suffer poorer HRQOL than patients in Western countries. Thus, identifying psychological factors is a top priority among Chinese BC patients.

The results of our study reported no association of sociodemographic and clinical variables with HRQOL among Chinese BC patients. However, other studies have suggested that SES indicators are uniquely associated with overall HRQOL and its dimensions in BC patients [9]. SES is defined as a multidimensional construct containing social position (education), economic status (income) and work conditions (occupation) [44]. For example, Xia et al. reported that financial difficulties were a stronger predictor of impaired global HRQOL for BC patients. You et al. also found a significant association of education and income with different aspects of HRQOL among BC patients [9].

The findings in our study presented a strong adverse effect of social constraints on HRQOL among Chinese BC patients, which was in accordance with the results in Caucasian [45] and Chinese American [24] patients with BC. Namely, patients with more social constraints tend to have worse HRQOL. Their abilities to perceive and ask for the social support they need and to express emotion are limited in a negative environment [25]. Finally, the way patients perceive their social environment seems to influence perceived stress [37]. For example, patients with a negative perception of their environment are more likely to consider a cancer diagnosis a more threatening, less manageable and stressful event, which results in adverse outcomes [37]. Additionally, our results revealed that social constraints among Chinese BC patients (Mean = 1.82) were higher than Caucasian BC patients (Mean = 1.77) in previous study [45], but were lower than those reported by Chinese American BC patients (Mean = 2.02) [24]. In Chinese culture, cancer is considered “bad luck”, and emotional expression is rare among Chinese families, which implies a high level of social constraints in Chinese BC patients. Hence, future research should adopt a cross-cultural design to directly compare the differences in social constraints in different countries.

Our study found that social support was positively associated with HRQOL, and the association of social support with HRQOL has been widely examined among BC patients [9, 15]. In addition, social support is beneficial for patients with different cancer types [46]. Patients with sufficient social support have a platform to share and communicate, which helps them adapt psychological changes and reconstruct cognitive processes. With regard to social stigma, the current study found that it was negatively related to HRQOL in BC patients. Previous research has also reported similar findings [14]. Some studies suggest that stigma may cause cognitive changes, including increases in negative thoughts and decreases in positive thoughts. These changes have an adverse influence on Chinese BC patients’ HRQOL. Therefore, psychological interventions to enhance the HRQOL of Chinese BC patients may attempt to foster social support and reduce social stigma and constraints.

### Implications

According to the findings from our study, some psychological interventions should be emphasized. Our study indicated the crucial psychological factors of HRQOL among Chinese BC patients. Our findings suggested that social constraints (e.g., emotional disclosure) and stigma are still common conditions in Chinese patients owing to traditional culture. Thus, intervention strategies focusing on less personal disclosure should be adopted. For instance, Chu et al. [47] found that expressive writing interventions effectively contribute to avoiding social constraints and decreasing posttraumatic stress disorder (PTSD) among Chinese American BC patients. Moreover, the findings of the current study acknowledge interventions based on positive social support to increase BC patients’ HRQOL. For example, families, friends and medical staff should immediately respond to their needs and questions in as many ways as possible.

### Limitations

Some limitations exist in the present study. First, this study had a cross-sectional design, and causal conclusions could not be drawn between social constraints, support, stigma and HRQOL among Chinese BC patients. Therefore, further research should adopt a longitudinal design to confirm these associations. Second, self-report surveys and convenience samples may limit representativeness in our study. Further studies with more representative samples should be performed. Third, the current study was performed in Liaoning Province and conducted in a small sample of patients. Thus, the participants in our study might not be representative of the population of BC patients. Finally, although our study found an adverse impact of social constraints on HRQOL among Chinese BC patients, the psychological pathways underlying this association are unclear. Future research should aim to explore the psychological pathways between social constraints and HRQOL.

## Conclusion

Our study is the first attempt to examine the relationship between social constraints and overall HRQOL among Chinese BC patients. The findings from the current study suggest that Chinese BC patients’ HRQOL needs to be enhanced after treatment. Social constraints have a strong association with HRQOL, while social support and stigma are also important factors. Given the above results, intervention strategies focusing on less personal disclosure should be considered to avoid social constraints and improve HRQOL among Chinese BC patients.

## Data Availability

The dataset in this study is available from the corresponding author on reasonable request.

## Abbreviations

BC: Breast cancer
HRQOL: Health-related quality of life
SD: Standard deviation
SES: Socioeconomic status
CNY: Chinese Yuan
FACT-B: Functional Assessment of Cancer Therapy-Breast
SCS-15: 15-Item Social Constraints Scale
MSPSS: Multidimensional Scale of Perceived Social Support
SIS: Social Impact scale
PWB: Physical well-being
SFWB: Social/family well-being
EWB: Emotional well-being
FWB: Functional well-being
BCS: Additional concerns
